# Intrauterine adhesions repair with menstrual blood-derived mesenchymal stem cells via CXCL13-CXCR5 signal axis and its mechanism

**DOI:** 10.1186/s13287-024-03996-7

**Published:** 2024-10-25

**Authors:** Bing Luo, Xun Zeng, Li Luo

**Affiliations:** 1 Department of Obstetrics and Gynecology, West China Second University Hospital, Sichuan University, Number 20, Third Section of People’s South Road, Chengdu, 610000 China; 2 Key Laboratory of Birth Defects and Related Diseases of Women and Children, Sichuan University, Ministry of Education, Chengdu, China

**Keywords:** Mesenchymal stem cells, Intrauterine adhesions, CXCL13- CXCR5 axis, Repair, Molecular mechanism

## Abstract

**Backgroud:**

Intrauterine Adhesions (IUA) is a common gynecological disease which is seriously endangers the reproductive function of women without any ideal treatment. Some researchers found Menstrual Blood-derived Mesenchymal Stem Cells (MenSCs) can repair of damaged endometrium, however, has not been fully clarified. This study aims to evaluate the therapeutic effects of MenSCs in IUA and the repair mechanism in vivo.

**Methods:**

This study is Laboratory-based study. To evaluate the therapeutic effects of MenSCs in IUA, We cultivated MenSCs, established mouse endometrial injury model, observed the uterine morphology and degree of endometrial fibrosis and compared the expression of CXC chemokine ligand-13 (CXCL13)、CXC chemokine receptor-5 (CXCR5)、Plasmin Activating Inhibitor-1(Pai-1), Transforming Growth Faction-β1(TGF- β1) and Matrix Metalloproteinase-9 (Mmp-9) among each groups. GraphPad Prism 8.0 was used for statistical processing. Data were expressed as mean ± SD. Statistical comparisons among groups were performed with one-way ANOVA. *P* < 0.05 were considered statistically significant.

**Results:**

We successfully cultured and identified MenSCs and established mice model of uterine adhesion. After treatment with MenSCs, endometrial morphology of mice was partially restored, endometrial thickness was increased, and glands were multipiled. The concentrations of CXCL13 and CXCR5 were significantly increased by immunofluorescence detection compared with the control group. The results of RT-qPCR showed that the expressions of Pai-1 and Mmp-9 were significantly lower than those of the control group.

**Conclusions:**

MenSCs may reduce endometrial fibrosis and the down-regulating expression of Pai-1、Mmp-9 and CXCL13-CXCR5 axis were involved in the process of MenSCs repaired IUA.

## Background

Intrauterine Adhesions (IUA), also known as Asherman’ s syndrome, is a common gynecological disease which is caused by destructive curettage or repeated endometritis and seriously endangers the reproductive function of women without any ideal treatment [1]. After impairment, the repair function of endometrial epithelial is damaged, the stroma is exposed, the fibroblasts proliferate excessively, which leads to vascular regeneration and endometrial proliferation disorder, and the deposition of fiber cells forms cicatrix, which leads to infertility in women [2]. Although hysteroscopic surgery can separate and cut off cicatrix tissue and restore the anatomical structure of uterine cavity, the basal layer of uterus is often seriously damaged, the uterus cannot repair itself, and the rate of postoperative re-adhesion is up to 62.5% [3].

It is, therefore, urgent to establish a new clinical treatment to repair the damaged uterus. Recent studies have shown that the repair ability of the endometrium is related to stem cells in the endometrium. In recent years, studies have shown that stem cell therapy, as a potential means of tissue and organ regeneration and repair, has attracted more and more attention. As a stem cell with multiple differentiation and high proliferation potential, Menstrual Blood-derived Mesenchymal Stem Cells (MenSCs) can be obtained from menstrual blood in many ways [4], boasting the advantages of reproducibility, non-invasiveness, little ethical argument and stable amplification in vitro without mutation. Some researchers found an increase in endometrial thickness and microvessels after implantation of MenSCs in the uterus of mice with endometrial injury [5], indicating that the endometrium can be partially recovered. The mechanism of MenSCs’ repair of damaged endometrium, however, has not been fully clarified.

CXCL13 is a multi-source cytokine, which is highly expressed in liver, spleen and lymph nodes. Its receptor CXCR5 can regulate the expression of inflammatory factors and participate in inflammatory response [6]. CXCL13 was first reported to be naturally expressed in human Mesenchymal stem cells (MSCs) [7]. Some researchers also found that CXCL13, CXCR5 may directly modulate cellular proliferation, migrate MSCs to the injured area, and induce differentiation of osteoblasts [8, 9]. yet its role in uterine adhesion is not clear.

This study aims at studying the repair effect of MenSCs and the role of CXCL13-CXCR5 axis in the treatment of IUA by MenSCs transplantation and its molecular mechanism, so as to provide new ideas for improving the efficacy of MenSCs in the treatment of IUA and the development of other MenSCs treatment regimens.

## Methods

### Laboratory animals

8-week-old sexually mature and unmated C57BL/6 female mice (SPF grade, Chengdu Lilai Biotechnology Co., Ltd, China) were used in the present study. They were housed three to four rats per cage at room temperature and given ad libitum access to a standardized diet and tap water. The content of this experiment involving animal experiments has been approved by the experimental Animal Management and Ethics Committee of West China Second Hospital of Sichuan University.

### Group and treatment

**Fig. 1:**
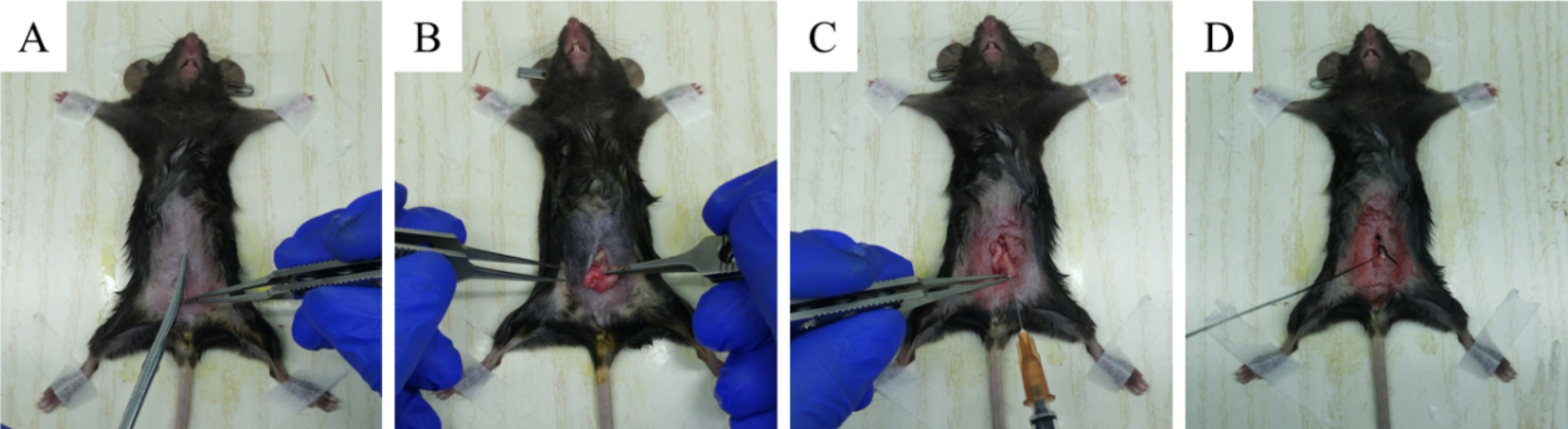
The schematic diagram for IUA modeling process. (**A**): A 1.5 cm longitudinal incision was made in the lower abdomen to expose the uterus; (**B**): Observe the morphology of the uterus and exclude mice with uterine abnormalities; (**C**): Scrape the uterine cavity in a clockwise direction with a uterine scraper; (**D**): Suture the abdominal skin and subcutaneous tissue

**Fig. 2:**
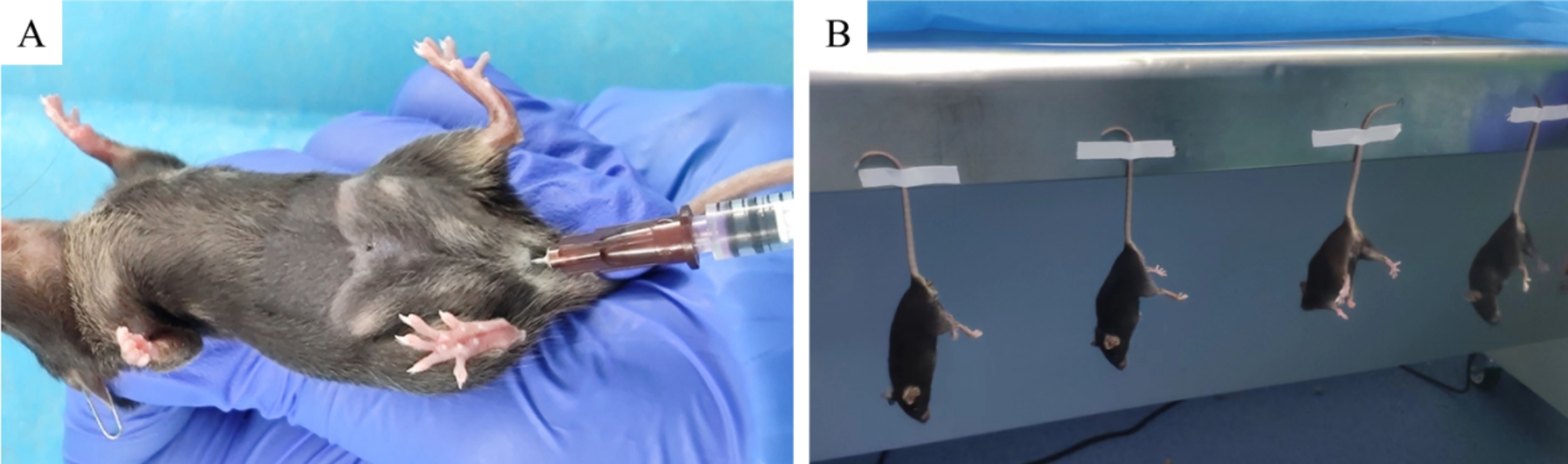
Transplantation treatment process. (**A**): intravaginal injection of MenSCs suspension; (**B**): post-administration treatment to prevent leakage

The mice were divided into 3 groups after being adaptively fed for 7 days: the control group (*n* = 7), open the abdominal cavity and pull out the uterus without other surgical treatment; IUA model group (*n* = 10), mechanical injure the uterine horns and inject 30 µl PBS via vagina at 7th, 11th, and 15th day after the operation and the process of IUA modeling was shown in Fig. 1; MenSCs treated group (*n* = 10), mechanical injure the uterine horns and inject 30 µl MenSCs suspension (1 × 10^7^/ml) via vagina at 7th, 11th, and 15th day after the operation. Four days after treatment, harvest the uterine tissues for morphological comparison and further examination. The specific grouping arrangements are shown in Table 1, and the transplantation treatment process is shown in Fig. 2. The mice were anesthetized by intraperitoneal injection of 30 mg/kg sodium pentobarbital before the experimental invasive procedure. After the end of the experiment, all the mice were anesthetized, and then the cervical vertebrae were dislocated and died.

**Table 1:** Grouping and administration of transplantation therapy

Group	Mouse	Quantity	Reagent	Dosage
Control group	Normal mice	7	1 × PBS	30 µ l
Model group	IUA model mice	10	1 × PBS	30 µ l
Stem cell group	IUA model mice	10	MenSCs suspension	30 µ l

**Table 2:** Primer sequence information

Genes	Primer sequence (5 ‘≤ 3’)
*Gapdh*	Upstream: AACATCATCCCTGCCTCTACTG
*Gapdh*	Downstream: TGCTTCACCACCTTCTTGATGT
*Pai-1*	Upstream: AGATGTCTTCAGCCCTTGCT
*Pai-1*	Downstream: CCATAGGGAGAGAAGACCAC
*TGF-β1*	Upstream: TGTGTGCGGCAGCTGTACAT
*TGF- β 1*	Downstream: TGTACTGTGTGTCCAGGCTC
*Mmp-9*	Upstream: TTGTACCGCTATGGTTACAC
*Mmp-9*	Downstream: TTGAAGGTTTGGAATCGACC

### Isolation and maintenance of MenSCs

Menstrual blood in the first four days was collected with a sterile menstrual cup and stored in a sterile tube with a preservation solution at 4℃. Inclusion criteria for menstrual blood are healthy females without vaginal discharge or infection, negative for HBV and HCV infection, and age ranges between 20 and 35 years old. Informed consent was obtained for every participant. 5 ml menstrual blood was decanted into an isolation buffer containing 2.5 µg/ml fungizone, 1% Pen/Strep, and 0.5 mM EDTA in 20 ml PBS. Then slowly added the blood sample into Ficoll-paque (No 17544202; Cytiva) of the same volume and centrifuged at 400 g for 20 min. The turbid interface phase of Ficoll and cell culture media was added in 3 ml PBS and centrifuged at 100 g for 10 min. The cell pellet was resuspended in DMEM-F12 (No. 10565018; Gibco), 10% FBS (No. 10099141c; Gibco), 1% Pene/Strep (No. 15140122; Gibco), 1% glutamine (No. 35050061; Gibco) at 37 ℃ in a 5% CO2 humidified incubator. The medium was refreshed every 2 days. Passaging was performed with 0.5% trypsin every 2 days when cell density reached 90%. Take the third-generation cells to record growth morphology and characterization.

### Flow cytometry

To confirm MenSCs identity, collect single-cell suspension using EDTA and resuspend them at a concentration of 1 × 10^7^ cells/ml in BD Pharmingen™ Stain Buffer (No. 554656; BD Bioscience). Cells were stained with a Human MSC Analysis Kit (No 562245. BD; USA) according to the manufacturer’s instructions and CD surface antigens were analyzed using a CytoFLEX LX (Beckman Coulter).

### Immunofluorescence

For MenSCs characterization, cells were plated on glass bottom culture dishes. When the cell density reached approximately 90%, MenSCs were fixed in 4% paraformaldehyde at 2–8℃ for 15 min and blocked with 10% goat serum for 30 min. Then, the cells were incubated with primary antibodies at 4℃ and in secondary antibodies for 1 h at room temperature. The antibodies used are as follows: Integrin beta 1 (ITGB1, No. ab179471; Abcam), CD105 (No. ab221675; Abcam), CD44 (No. ab243894; Abcam), CD90 (No. ab307736; Abcam), and Goat anti-Rabbit Alexa Fluor™ 555 (No. A32732; Invitrogen). After washing with PBS, incubate the cells with DAPI and cover them with an antifade mounting medium (No. H-1000; VECTASHIELD). For endometrial markers detection, each group of uterine tissue, after fixation and dehydration, was embedded in OCT and cut into sections of 10 μm in thickness. After washing with PBS, the slices were blocked with 10% goat serum and 0.1% Triton X-100 in PBS at room temperature for 1 h. After block and permeabilization, the sections were immersed in anti-Cxcl13 (No. 15782; ABclonal) and anti-Cxcr5 (No. A8950; ABclonal) at 2–8℃ overnight. Goat anti-Rabbit Alexa Fluor™ 555 (No. A32732; Invitrogen) was incubated with cells at room temperature for 1 h. A confocal microscope (Leica SP8) was used to collect the images. The fluorescence intensity was analyzed by ImageJ software.

### HE staining

A segment of the uterus in the same position was taken and fixed in 4% paraformaldehyde for more than 24 h, embedded in paraffin, and sliced. Xylene dewaxed for 5 min, dehydrated with different concentrations of ethanol, stained with hematoxylin staining solution for 5 min, differentiated for 30 s, soaked in 1 × PBS for 15 min, soaked in eosin for 1 min, dehydrated with different concentrations of ethanol, treated with xylene transparent and fixed with paraffin. The morphology of the endometrium was observed under a microscope, and the thickness of the endometrium, the number of glands and endometrial blood vessels were calculated with Image pro plus 6.0.

### Masson staining

The sections were conventional dewaxed and stained with Weigert ferritin dye, ponceau acid fuchsin staining solution, and Aniline blue staining solution, respectively. Different concentrations of ethanol were used for dehydration and xylene transparent treatment. After fixation, the blue staining area ratio of endometrial fibrosis was calculated by Image Pro plus 6.0 under a microscope.

### RT-qPCR

MiRNeasy Mini Kit (No. 160040742; QIAGEN) was used to extract total RNA from the uterus. RNA concentration and purity were measured by NanoDrop 2000 C spectrophotometer. The total RNA samples were reversely transcribed using a cDNA Synthesis Kit (No. R211-01; Vazyme). The amplification system was prepared according to the specification step by Taq Pro Universal SYBR qPCR Master Mix (No. Q712-02; Vazyme). The dissolution curve and CT value results were derived after amplification, and three experiments were carried out repeatedly. The relative gene expression levels were calculated using the 2^−△△CT^ method. The primers used for the RT-qPCR analysis are presented in Table 2.

### Statistical analysis

GraphPad Prism 8.0 was used for statistical processing. Data were expressed as mean ± SD. Statistical comparisons among groups were performed with one-way ANOVA. *P* < 0.05 were considered statistically significant. The work has been reported in line with the ARRIVE guidelines 2.0.

## Results

### Morphological observation and surface marker identification of MenSCs

**Fig. 3:**
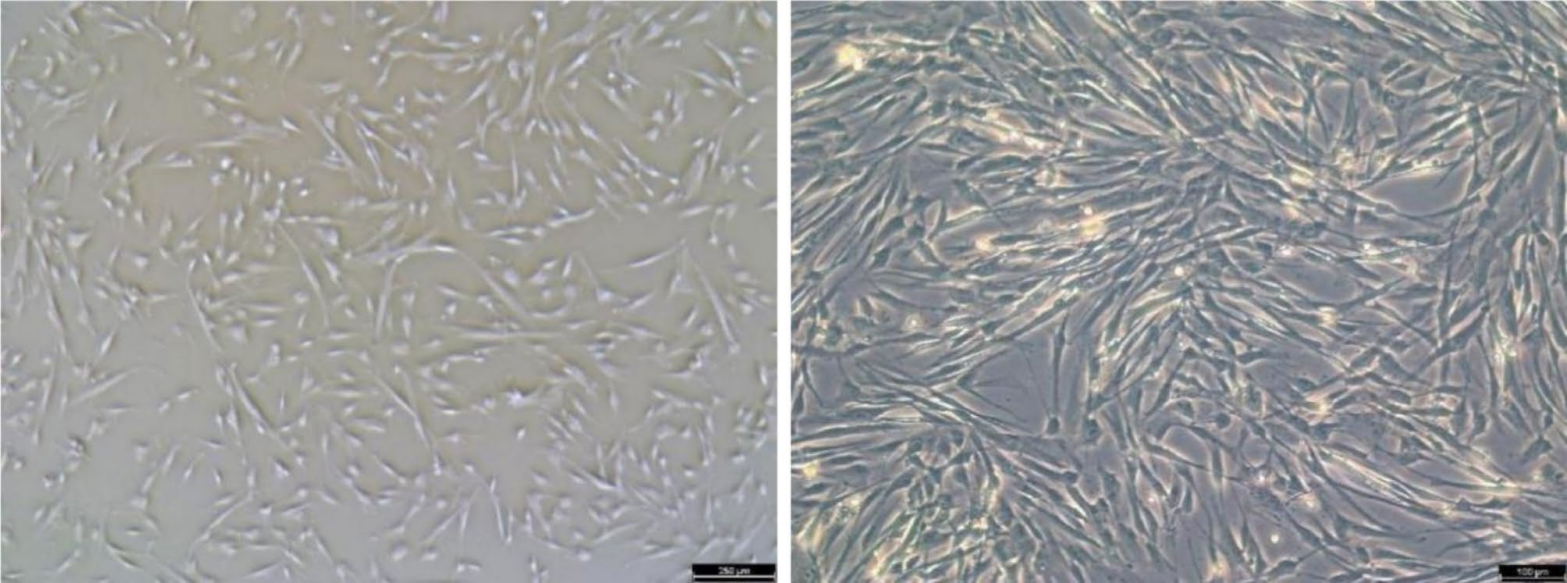
Morphology of MenSCs under inverted phase contrast microscope (left 40 ×, right 100 ×)

After 24 h of MenSCs resuscitation, the cells began to adhere to the wall, mostly in triangular or irregular shape, and the un-adherent cells were discarded in 72 h, and the cells formed colonies of different sizes. After that, the number of adherent cells increased gradually, with the characteristics of fibroblasts in uniform fusiform distribution, and vortex-like adherent growth at 5 days (see Fig. 3).

**Fig. 4:**
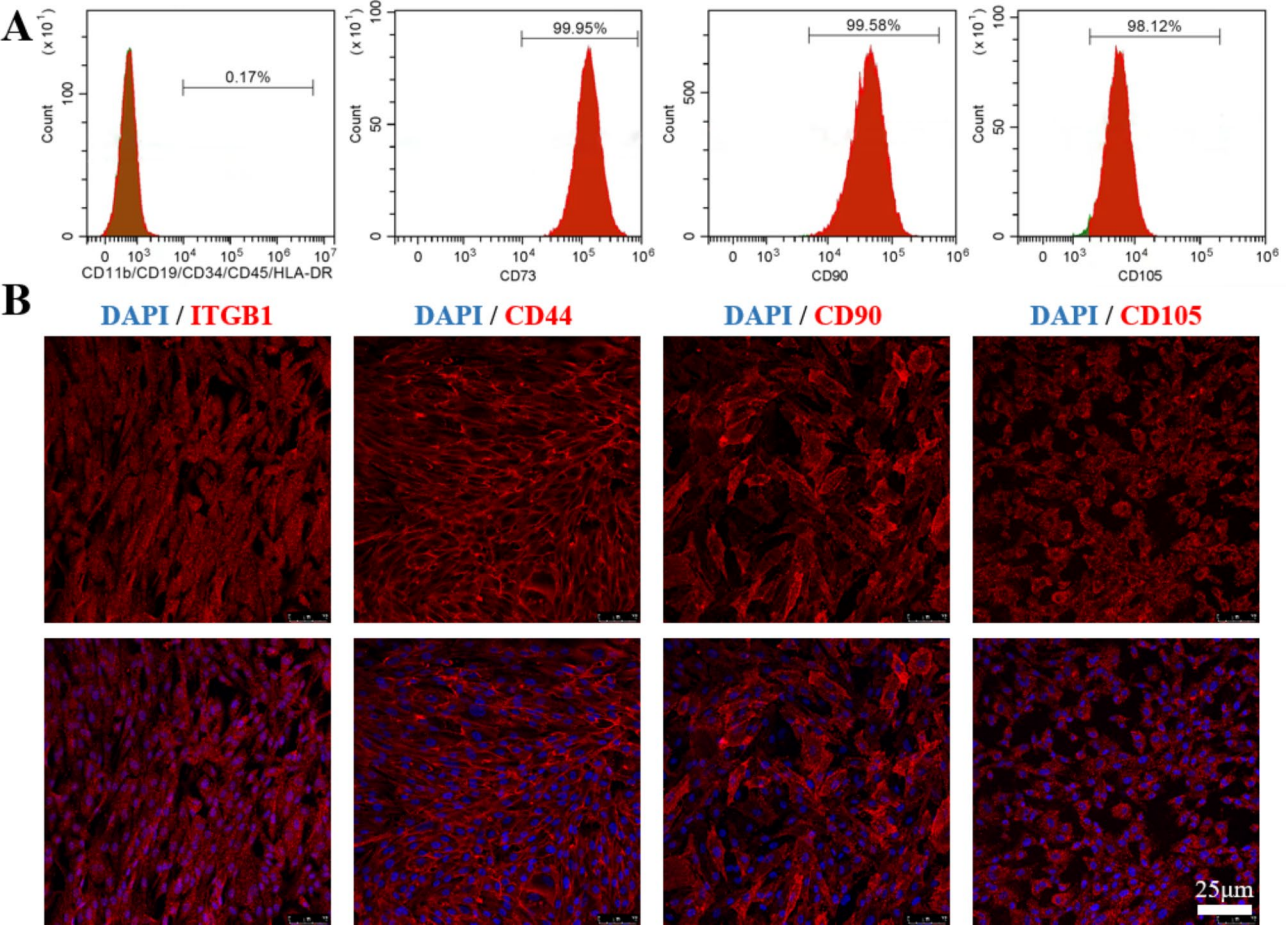
Immunophenotypic characterization of MenSCs. (**A**) Flow cytometry characterization analyses of the MenSCs showed uniformly positive expression for CD73, CD90, CD105 while negative for CD11b, CD19, CD34, CD45, HLA-DR. (**B**) Immunostaining for Integrin beta 1 (ITGB1), CD44, CD90, and CD105 of MenSCs. Scale bar, 25 μm

To determine the immunophenotype, MenSCs were stained by corresponding conjugated antibodies and analysed by flow cytometry. The results indicated that the isolated cells reproduced the positive phenotype of MenSC (CD73, CD90, CD105) and lacked of negative surface markers (CD11b, CD19, CD34, CD45, HLA-DR) (Fig. 4. A). The immunofluorescence also confirmed this result, as MenSCs are positive for ITGB1, CD44, CD90, and CD105 (Fig. 4. B).

### Anatomical morphology of mice uterus

**Fig. 5:**
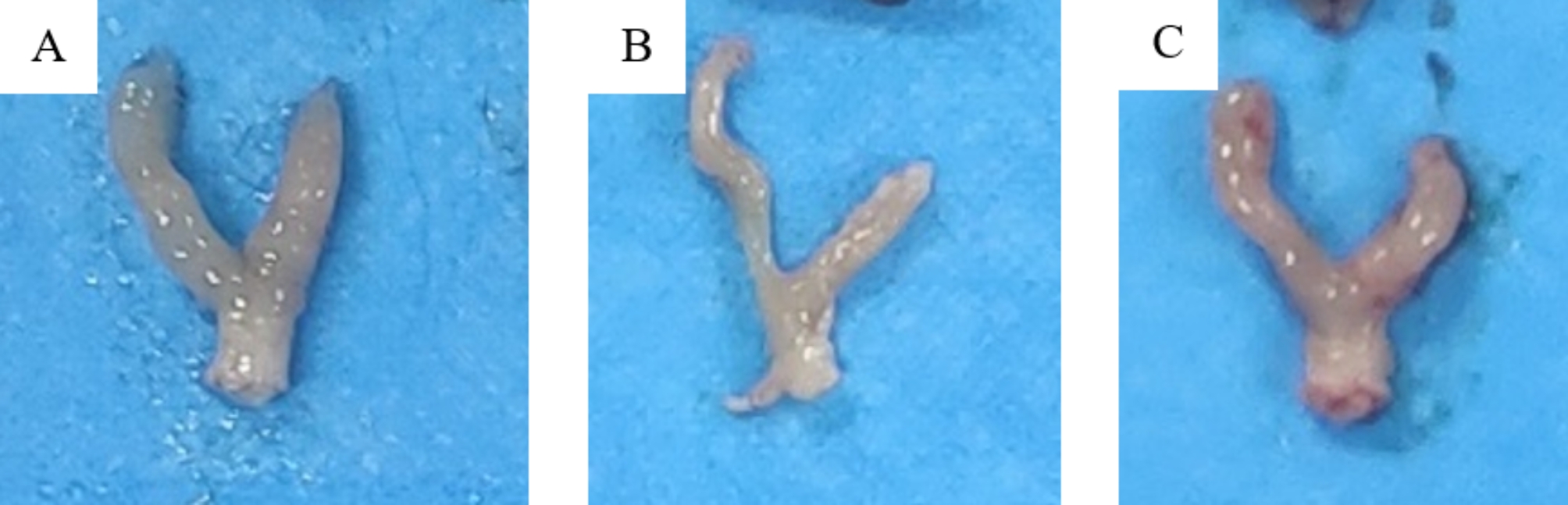
Uterine morphology of mice in each group. (**A**): control group; (**B**): model group; (**C**): stem cell group

The anatomical morphology of uterus in each group was shown in Fig. 5. Compared with the control group, the uterus of the model group was irregular and elastic, and adhesion could be seen. After transplantation of MenSCs, most of the uterine cavity morphology returned to normal, and a few of the uterine cavity slightly adhered. It was indicated that MenSCs transplantation can restore the uterine structure of uterine cavity adhesion to a certain extent.

### HE staining results

**Fig. 6:**
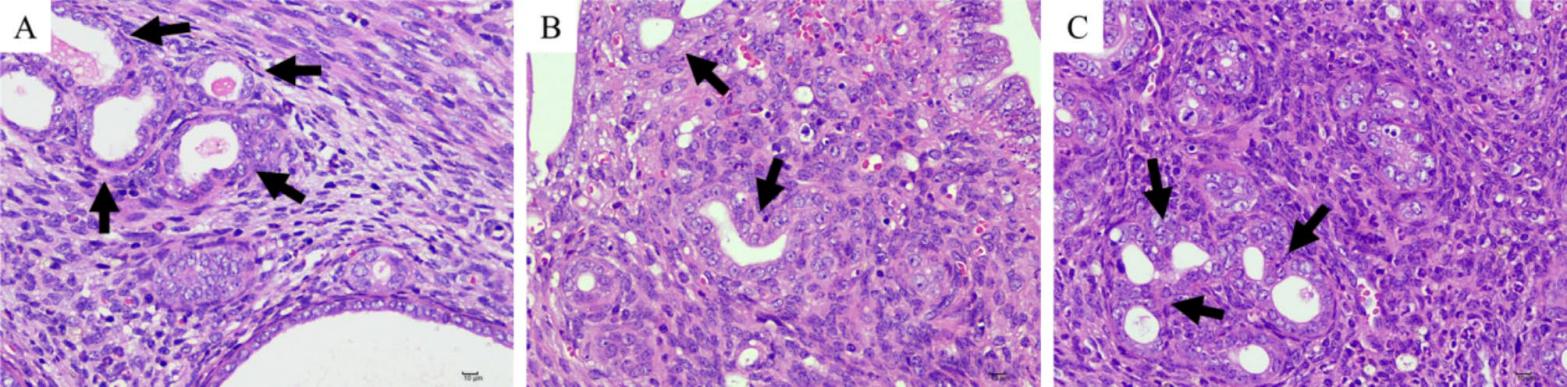
HE staining in uterus of mice in each group (400 ×). (**A**): control group; (**B**): model group; (**C**): stem cell group

**Fig. 7:**
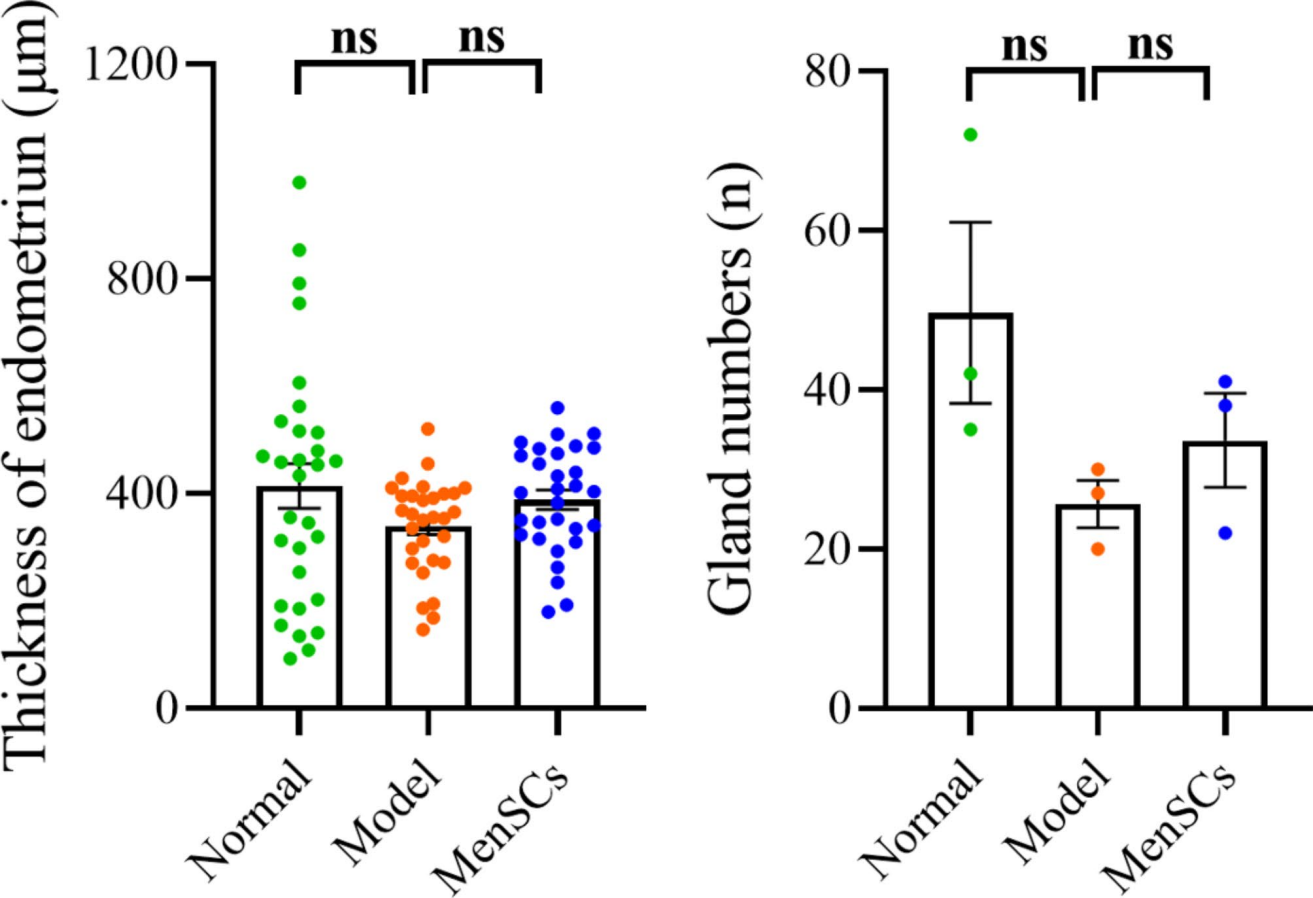
Thickness of endometrium and number of uterine glands in each group of mice

The results of HE staining were as follows: Fig. 6. The endometrial structure of the control group was clear and complete, the gland was rich in round or oval shape, compared with the control group, the endometrium of the model group was thinner or missing, the number of glands were significantly decreased, and the gland cavity was dilated or closed. After transplantation of MenSCs, the endometrial structure of the mouse recovered, the number of glands increased, regular, round or oval (see Fig. 7).

### Masson staining results

**Fig. 8:**
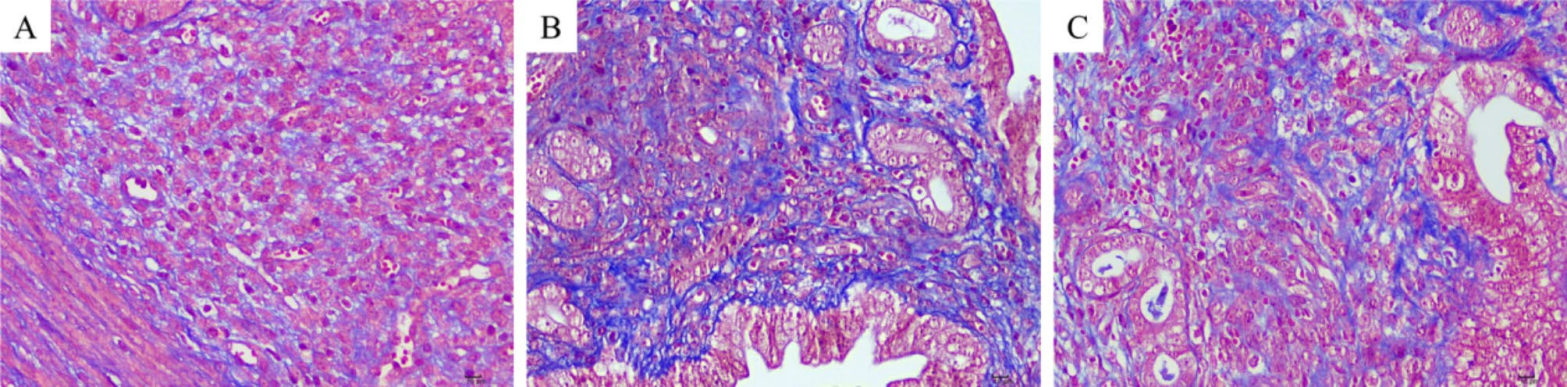
Masson staining in uteruses of mice in each group (400 ×). (**A**): control group; (**B**): model group; (**C**): stem cell group

**Fig. 9:**
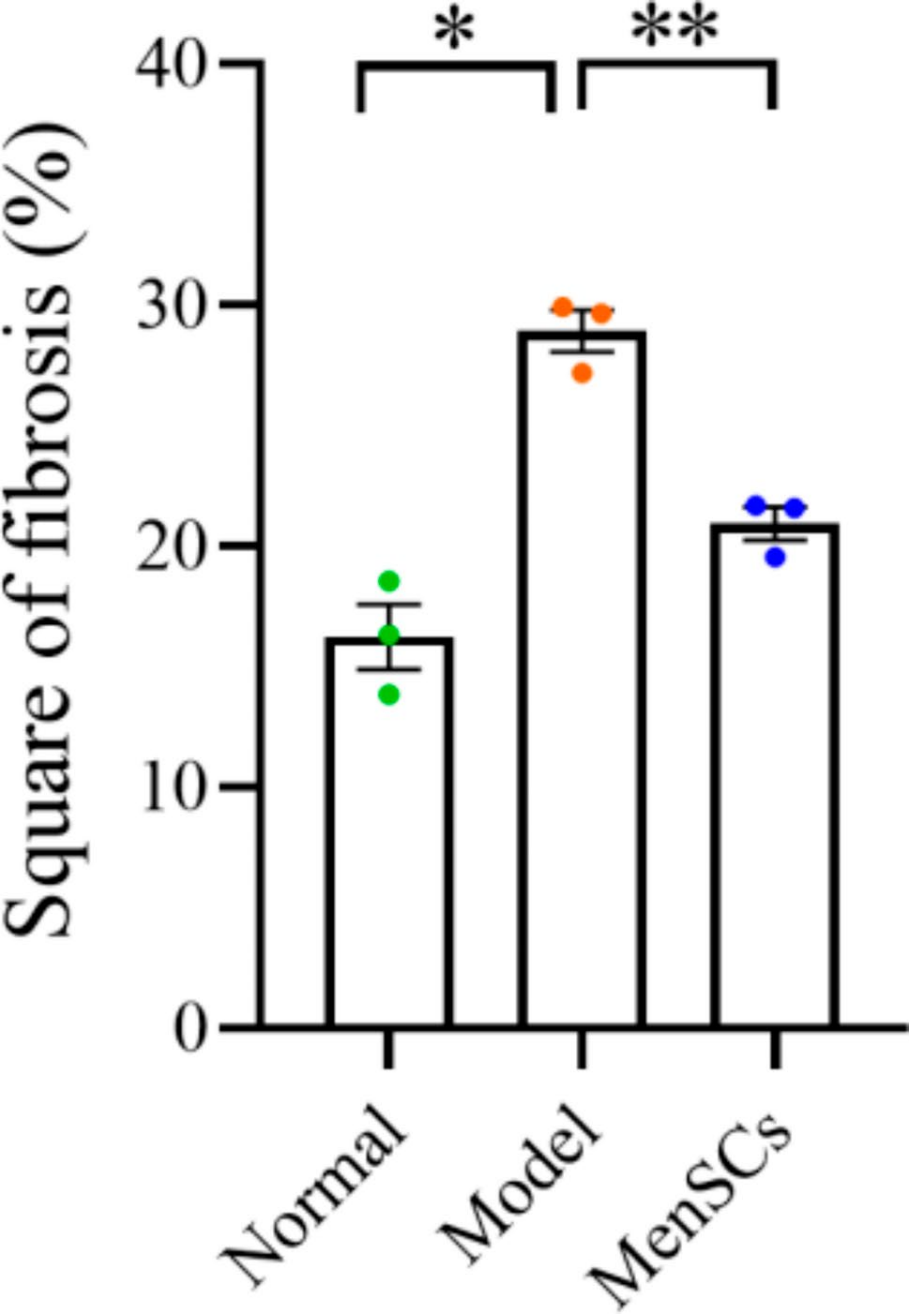
Ratio of endometrial fibrosis area in each group of mice

The results of Masson staining were shown in Fig. 8 in the control group, slight loose light blue fiber was found in the control group, compared with the control group, a large number of dense and deeply stained blue fibers were found in the uterus of the model group, and a medium amount of loose blue fiber could be seen in the uterus after transplantation of MenSCs. The results showed that MenSCs transplantation could alleviate the fibrosis degree of uterine adhesion in mice. According to the statistical analysis of the area ratio of endometrial fibrosis in each group, the degree of fibrosis in the IUA model group was significantly increased, and the degree of fibrosis in the IUA model group was significantly lower than that in the model group after stem cell treatment (see Fig. 9).

### Immunofluorescence results

**Fig. 10:**
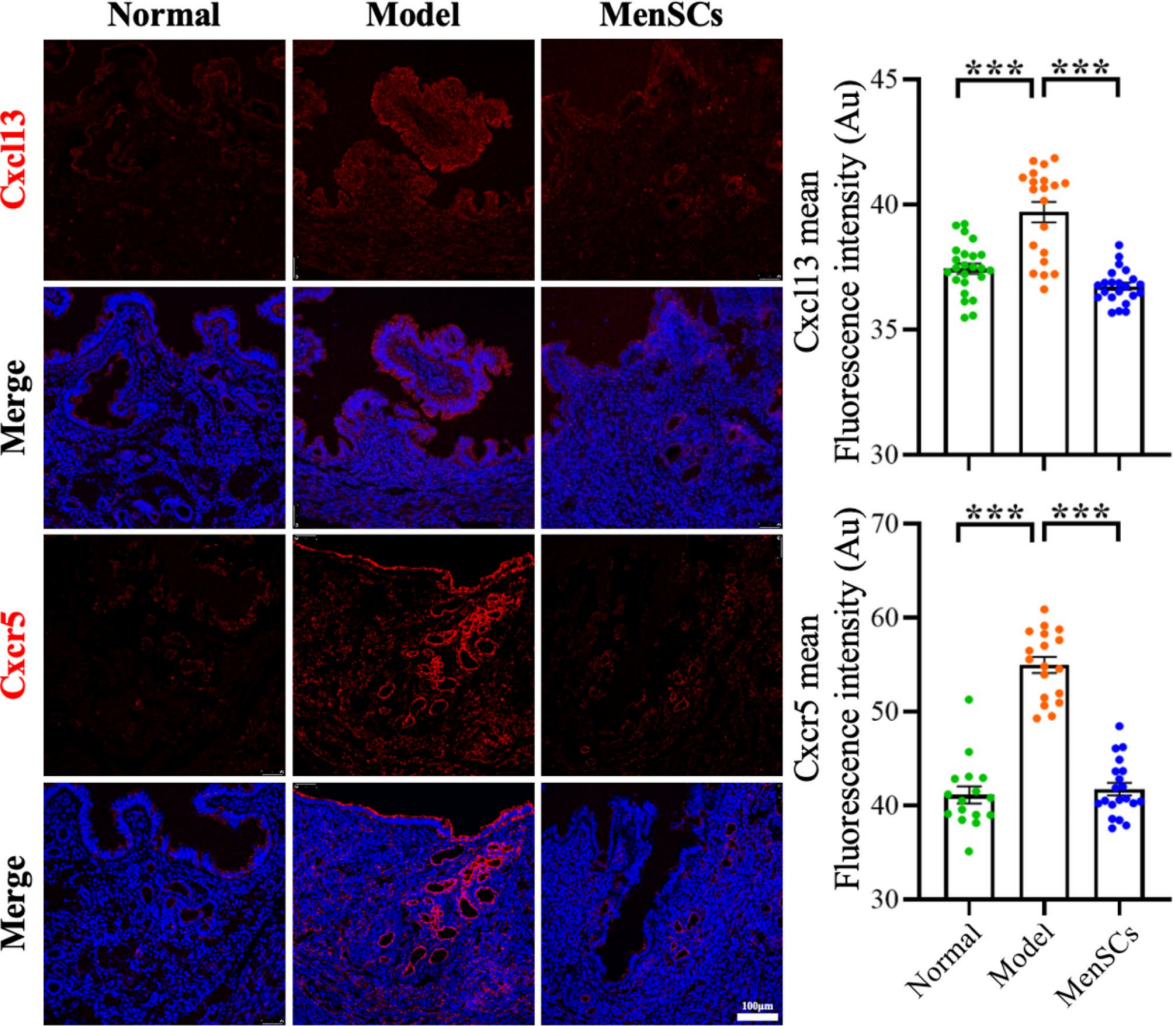
Expression level of Cxcl13 and Cxcr5

Compared with the control group, the fluorescence density of CXCL13 and CXCR5 in the IUA model group was significantly higher than that in the control group, and the fluorescence density in the stem cell group was significantly lower than that in the control group (see Fig. 10).

### RT-qPCR result

**Fig. 11:**
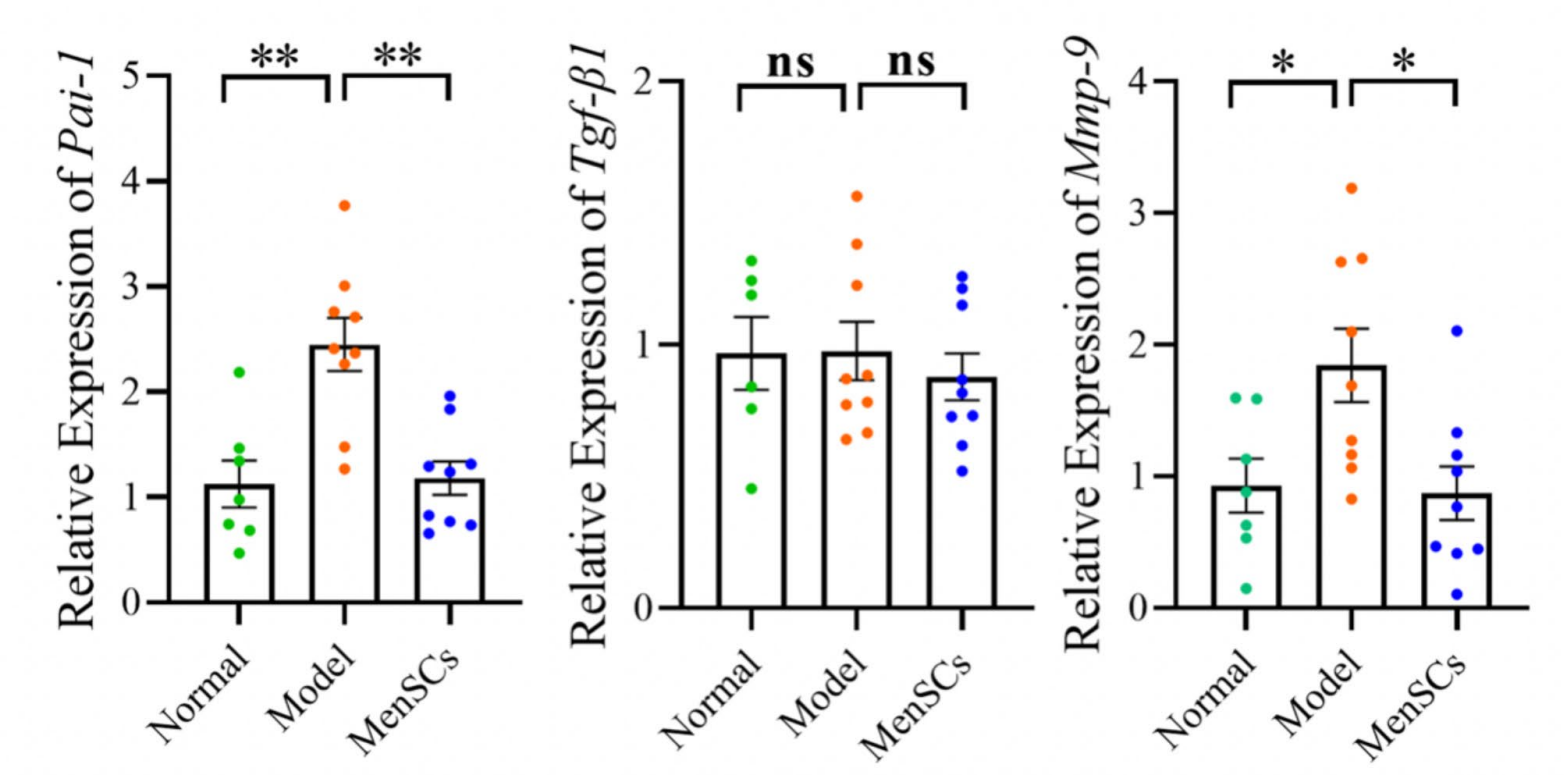
mRNA expression levels of *Pai-1*, *TGF- β 1* and *Mmp-9*

It is known that Pai-1, TGF-β1 and Mmp-9 can inhibit the degradation of ECM, and their expression levels are positively correlated with endometrial fibrosis. The results showed that compared with the control group, the relative expression levels of Pai-1 and Mmp-9 in the IUA model group were significantly higher and the expression level in the stem cell group was significantly lower than that in the model group. There was no significant difference in the expression level of TGF- β 1 among the three groups (see Fig. 11). It is suggested that the effect of MenSCs in the treatment of uterine adhesions may decrease the level of endometrial fibrosis by regulating the expression of Pai-1 and Mmp-9.

## Discussion

Multiple studies have shown that in mouse models of uterine adhesion, endometrial thickness and pregnancy rate could be improved to a certain extent after bone marrow mesenchymal stem cells(BMSCS)treatment by caudal vein or orthotopic transplantation of BMSCS into the damaged uterine cavity [10–12]. Taylor et al. reported that endometrial cells differentiated from donor BMSCS were found in the uterus after BMSCS transplantation, suggesting that BMSCS may play an important role in endometrial reconstruction [13]. Due to the invasive operation of BMSCS and unstable donors, however, the widespread use of BMSCS in clinical practice is limited.

MenSCs could be obtained from menstrual blood with the advantages of repeatability, non-invasiveness and few ethical controversies [4]. The therapeutic potential and mechanism of MenSCs have received increasing attention from researchers. Recently, after implantation of MenSCs in the uterus of a mouse model with endometrial injury, researchers found that the endometrial thickness and microvessels added, suggesting that the endometrium was partially restored [5]. It has also been reported that MenSCs can repair endometrial stromal cells by activating P38 MARK and AKT signaling pathways, and promote angiogenesis and repair endometrial through ERK pathways [5, 14]. A clinical case [15] reported that MenSCs were transplanted into the uterus of the patient with severe intrauterine adhesion, and the endometrium reached the minimum standard of embryo implantation after the treatment, and the patient finally succeeded in conception through assisted reproductive technology, suggesting that MenSCs plays an important role in endometrial stromal cell repair and endometrial function restoration, but the specific biological mechanism needs to be further studied. We, therefore, cultivated MenSCs and established a mouse endometrial injury model by a mechanical method to evaluate the therapeutic effects of MenSCs.

By observing the uterine morphology of mice, we compared the model groups and found that the uterine cavity morphology of mice after MenSCs transplantation was mostly restore and the uterine cavity was only slightly adherent, indicating that MenSCs could restore the uterine adhesion structure to a certain extent. As endometrial thickness and number of glands significantly affect clinical pregnancy and live birth rate in embryo transfer cycle [16], we measured the endometrial thickness and number of glands in our animal study. In our mouse uterine adhesion model, we found that the MenSCs-treated mice had remarkable endometrial thickness improvement after MenSCs injection. There was, however, no significant difference in the number of glands. In our study, Masson staining was performed on the endometrium of each group to observe the degree of endometrial fibrosis, and it was found that a large number of dense blue fibers was observed in the endometrium of the IUA group, while a moderate amount of loose blue fibers was observed in the endometrium after MenSCs transplantation, indicating that MenSCs treatment could alleviate the degree of endometrial fibrosis.

CXCL13, also known as B-lymphocyte chemical attractant (BCA-1), is a polygenic cytokine highly expressed in liver, spleen, and lymph nodes, and is capable of attracting B cells with the receptor of CXCR5, which is of great significance to promote the proliferation and migration of MSCs via PI3K/AKT signaling pathway under hypoxia conditions of tissue injury, and to promote the repair of tissue injury [6]. Studies on the correlation between CXCL13 /CXCR5 signaling axis and gastric cancer, breast cancer, lung cancer and other tumors [17–20] have been reported, but the correlation of CXCL13 /CXCR5 signaling axis in uterine adhesions remains unclear. CXCL13 is one of the most abundant chemokines in endometrial epithelial cells [21]. Kitaya found the aberrant expression of CXCL13 in the endometrium of patients with chronic endometritis [22]. In this study, it was also found that the expressions of CXCL13 and CXCR5 in the endometrium of IUA mice group were significantly higher than those in the control group and CXCL13 and CXCR5 in endometrium of MenSCs mice group were significantly lower than IUA group, so it is inferred that MenSCs treatment could reduce the expression level of CXCL13 and CXCR5 in the endometrium. Studies have shown that CXCL13 is involved in the PI3K/AKT signaling pathway to regulate the expression of Mmp-9, Pai-1 and βTGF-β1 [23].

Mmp-9 is a group of hydrolytic enzymes that can degrade type IV and V collagen. High levels of Mmp-9, however, may cause damage to the endometrial histological barrier. Studies have shown that the positive expression rate of Mmp-9 in endometrial tissue of patients with intrauterine adhesion group is higher than that of control group. The overexpression of Mmp-9 may induce EMT process and promote the progression of fibrotic disease. Our experimental results also found that Mmp-9 in endometrial tissue of patients with uterine adhesion group was higher than that of control group and MenSCs group. TGF-β1 is currently recognized as the strongest pro-fibrotic factor which is closely related to the formation of uterine adhesions. Pai-1 is a physiological inhibitor of fibrinolysis system, which could inhibit the degradation of extracellular matrix (ECM), promote the accumulation of extracellular matrix and aggravate the degree of endometrial fibrosis by inhibiting the activity of tissue polypeptide antigen. Our experimental results showed that the expression level of Pai-1 in endometrium of MenSCs group was significantly lower than that of control group and IUA group, while TGF-β1 was not significantly different among all groups. These results indicate that MenSCs may reduce endometrial fibrosis by regulating the expression of Pai-1 and Mmp-9, but the specific mechanism was unclear. It may be regulated by relevant pathways, and thus needs to be further verified.

## Conclusions

In conclusion, our study revealed that endometrial morphology of IUA mice was partially restored, endometrial thickness was increased, and glands were multiplied after MenSCs treatment. In vivo experiments suggested that the down-regulating expression of Pai-1, Mmp-9 and CXCL13 /CXCR5 signaling axis were involved in the process of MenSCs repaired IUA. The current findings offered a theoretical basis for better application of MenSCs to IUA healing and implied that CXCL13 /CXCR5 signaling axis maybe a potential molecular direction in IUA repaired, but the specific mechanism awaits further study.

## Data Availability

All data and materials relevant to this study are included in the article, and further inquiries can be directed to the corresponding author.

## Abbreviations

Intrauterine Adhesions: IUA
Menstrual Blood-derived Mesenchymal Stem Cells: MenSCs
Mesenchymal stem cells: MSCs
bone marrow mesenchymal stem cells: BMSCS Metalloproteinase-9:Mmp-9
Plasmin Activating Inhibitor-1: PAI-1
Transforming Growth Faction-β1: TGF-β1
extracellular matrix: ECM
